# Are the Risk Factors for Developing Type 1 and Type 2 Cesarean Scar Pregnancy Different?

**DOI:** 10.1002/jum.16662

**Published:** 2025-02-12

**Authors:** Yunhui Tang, Cuifeng Qian, Man Li, Min Zhao, Qi Chen

**Affiliations:** ^1^ Department of Family Planning The Hospital of Obstetrics and Gynaecology, Fudan University Shanghai China; ^2^ Department of Ultrasound The Hospital of Obstetrics and Gynaecology, Fudan University Shanghai China; ^3^ Department of Gynaecology Wuxi Maternity and Child Health Hospital, Wuxi Medical School, Jiangnan University Wuxi China; ^4^ Department of Obstetrics and Gynaecology The University of Auckland Auckland New Zealand

**Keywords:** cesarean scar pregnancy, clinical risk factors, previous cesarean sections, type 1 CSP, type 2 CSP

## Abstract

**Objectives:**

Cesarean scar pregnancy (CSP) is a life‐threatening complication of pregnancy. Early termination of pregnancy is recommended because of the potential risks of uterine rupture if the pregnancy continues. CSP is clinically divided into type 1 and type 2, based on the site of embryo implantation and grow direction of the gestational sac. The clinical risk and treatment options may vary between these subtypes. Identifying the differences and potential factors impacting the development of each type could contribute to improved clinical management.

**Methods:**

This retrospective study included 143 women with type 1 CSP and 200 women with type 2 CSP women, diagnosed between January 2020 and July 2023. The primary objective was to analyze the differences in clinical characteristics between the 2 CSP subtypes.

**Results:**

Body mass index, the number of previous CSPs, elective abortions, gravidity, and parity were not significantly different between the 2 subtypes. Similarly, the interval between the last cesarean section and the current CSP was not associated with developing the 2 subtypes. However, women with more than 3 prior cesarean sections had an increased risk of developing type 1 CSP. In contrast, vaginal bleeding before diagnosis was more frequently associated with type 2 CSP.

**Conclusions:**

Our study with a large sample size found that despite type 2 CSP involving deeper myometrial invasion, the 2 subtypes share many clinical characteristics. Moreover, the clinical risk factors for distinguishing between type 1 and type 2 CSP are limited. Future research to identify predictors for CSP classification is required.

**Abbreviations**
BMI: body weight index
CSP: cesarean scar pregnancy
hCG: human chorionic gonadotropin
SD: standard deviation

A significant increase in cesarean sections worldwide, in particular in China, with the highest cesarean section rate,^1^ results in an increase in the incidence of cesarean scar pregnancy (CSP) in China.^2^, ^3^ CSP affects approximately 0.2% of women with a history of cesarean section,^4^ and is a rare form of ectopic pregnancy where the embryo fully or partially implants either in the muscle or fibrous tissue of the scar after a previous cesarean section.^5^ CSP carries a high risk of uterine rupture and life‐threatening bleeding if the pregnancy continues.^6^, ^7^ Therefore, the termination of the pregnancy is clinically recommended upon confirmation of diagnosis.

Based on imaging findings and pregnancy progression, CSP is traditionally classified into 2 subtypes. Type 1 (or endogenic) CSP is defined when the embryo implantation is located on the site of the scar, and the gestational sac grows toward the uterine cavity. Type 2 (or exogenic) CSP is defined when the embryo is implanted into the scar, and the sac grows toward the abdominal cavity (^7^, ^8^, ^9^). An urgent need to develop screening and management recommendations to better understand the risks associated with CSP.^10^ The 2 subtypes of CSP could manifest in different complications. For example, uterine rupture occurs more often in type 2 CSP (review in Shafqat et al^11^). Additionally, there is no universally accepted standard option for managing 2 types of CSP,^9^, ^12^ and optimal management may differ between the 2 types of CSP, and that would depend on the experiences of gynecologists. However, emerging evidence suggests that subtype‐specific approaches might be necessary because the severity and risks can vary significantly between subtypes.

Although the gold standard for diagnosis of CSP is ultrasound or magnetic resonance imaging by visualizing the gestational sac's location in the diagnosis of CSP,^13^, ^14^ whether there is a difference in clinical risk factors between women who developed type 1 and those who developed type 2 CSP has not been fully investigated. The development of type 1 or type 2 CSP can be influenced by various risk factors, including anatomical, physiological, and possibly genetic factors. Some studies have suggested that several factors including the location of the uterine scar from a previous cesarean section or other uterine surgeries, the number of prior cesarean sections, the depth of scar tissue from the last cesarean section, surgical techniques, healing response, and hormonal and genetic factors, may contribute to the development of different types of CSP. Women with multiple cesarean sections may have more extensive scar tissue, and this effect could increase the risk of developing type 1 CSP.^15^ However, to date, it still needs to be clarified why 1 type of CSP occurs in some women while the other occurs in others.

Understanding these differences is essential for advancing preventive strategies and creating uniform treatment protocols, which are currently lacking. Therefore, we undertook this study with a large sample size to investigate the differences in the clinical risk factors between women who developed type 1 CSP and those who developed type 2 CSP. The primary aim of this study is to understand the differences in risk factors in developing different types of CSP.

## Methods

Data for this study were collected prospectively during a previous investigation conducted from January 2020 to July 2023. The Ethics Committee of The Hospital of Obstetrics and Gynaecology, Fudan University, China approved the original prospective study (reference number: KYY2020‐185 and KYY 2020‐11).

### Study Cohort

During the study period from January 2020 to July 2023, a total of 343 women diagnosed with CSP who were prospectively recruited from our hospital clinics, were included in this study. CSP was confirmed by ultrasound, showing the presence of a gestational sac in the area of the cesarean scar, along with a history of a prior cesarean section and a positive pregnancy test. Based on the image findings detected by ultrasound, the CSP was divided into 2 subtypes. Type 1 CSP is defined when the embryo is implanted in the myometrium and grows toward the cervico‐isthmic space or uterine cavity. Type 2 CSP is defined when the embryo is implanted deeply within the scar tissue and penetrates the uterine wall, sometimes reaching the uterine blood vessels, as determined by ultrasound.^9^, ^16^, ^17^

The clinical risk factors were collected from the hospital's electronic database. Data included maternal age, body weight index (BMI), clinical symptoms such as vaginal bleeding, parity and gravidity, gestational age, the number of surgical abortions, the previous CSP, the number of prior cesarean sections, the interval between the last cesarean section and the current CSP, and the serum levels of β‐human chorionic gonadotropin (hCG). Additionally, the information on pregnancy mood swings was self‐reported.

The primary outcome was to analyze the differences in clinical characteristics as risk factors between the 2 subtypes of CSP.

### Statistical Analysis

Data were presented as mean, median, standard deviation (SD), range, or percentage when appropriate. T‐test (Mann Whitney test), Fisher's exact test, or chi‐square test were performed for statistical analysis using GraphPad Prism (version 10.1.2). This study was primarily designed to describe the distribution and characteristics of CSP cases. However, we then extended the analysis to examine potential associations between variables between the 2 CSP subtypes based on clinical characteristics. Statistical significance was considered when the *P* < .05.

## Results

In total of 343 women diagnosed with CSP included in this study, 143 (42%) women were diagnosed with type 1, and 200 (58%) women were diagnosed with type 2 CSP. The comparison of clinical characteristics between type 1 CSP and type 2 CSP is summarized in Table 1. There was no statistical difference in maternal age at diagnosis (35 versus 35 years, *P* = .074), in BMI at diagnosis (22.4 versus 22.8 kg/m^2^, *P* = .888), and in women who experienced previous CSP between the 2 groups (6.7% versus 3.5%, *P* = .226, Table 1). While 63% of women with type 2 CSP experienced vaginal bleeding before treatment, which was significantly higher than those women with type 1 CSP (52% versus 64%, *P* = .04, Table 1). The gestational age in type 2 CSP was significantly greater than that in type 1 CSP (*P* = .028). In addition, there was no statistical significance in levels of β‐hCG between the 2 types of CSP (*P* = .252).

**Table 1:** General Clinical Characteristics in the Study Cohort

	Type 1 (n = 143)	Type 2 (n = 200)	*P* Value
Maternal age at the time of diagnosis (years, mean/SD)	35 ± 4.5	35 ± 4.1	.074
Gestational age (days, men/SD)	49 ± 11	51 ± 11	.**028**
BMI (kg/m^2^, mean/SD)	22.43 ± 3.6	22.78 ± 3.6	.888
Vaginal bleeding, n (%)			
Yes	75 (52.4%)	127 (63.5%)	.**040**
Women with a previous CSP, n (%)			
Yes	9 (6.7%)	7 (3.5%)	.226
No	134 (93.7%)	193 (96.5%)	
β‐hCG (mIU/mL, median/range)	29835 (223 to 12268469)	37057 (41 to 286359)	.2523

*Note*: Values in bold indicate statistical significance (*P* < .05).

We then compared the pregnancy history between women with type 1 and type 2 CSP. There was no statistical significance in gravidity, parity, and the number of abortions between type 1 and type 2 CSP (Table 2, *P* > .05). There was also no statistical significance in the interval between the last cesarean section and the current pregnancy (Table 2, *P* = .928). There was no statistical significance between the 2 groups of CSP women with twice the previous cesarean section (*P* = .740). However, the increased cesarean sections (more than 3 times) significantly increased the risk for developed type 1 CSP (*P* = .038). The odds ratio for women with more than 3 previous cesarean sections who developed type 1 CSP was 4.410 (95% CI: 1.184–15.31, Table 3) compared to women who developed type 2 CSP. However, there was no statistical difference in CSP women with 2 prior cesarean section between the 2 types of CSP (*P* = .2168). In addition, a significantly higher percentage of women with a history of hysteroscopic surgery was observed in women with type 1 CSP compared to those with type 2 CSP (6% versus 0.5%, *P* = .005).

**Table 2:** Summary of the History of Pregnancies

	Type 1 (n = 143)	Type 2 (n = 200)	*P* Value
Gravidity, n (%)			
G = 1	1 (0.4)	0 (0)	.278
G = 2	14 (8.7)	11 (5.5)	
G = 3	31 (21.5)	49 (24.5)	
G ≥ 4	97 (69.4)	140 (70)	
Parity, n (%)			
P = 1	77 (53.8)	109 (54.5)	.**046**
P = 2	54 (37.8)	86 (43.0)	
P ≥ 3	12 (8.4)	5 (2.5)	
Abortion, n (%)			
A = 0	20 (14.0)	23 (11.5)	.399
A = 1	43 (30.1)	65 (32.5)	
A = 2	49 (34.2)	56 (28.0)	
A ≥ 3	31 (21.7)	56 (28.0)	
Previous CS, n (%)			
Once	85 (59.4)	115 (57.5)	.**038**
Twice	49 (34.3)	82 (41.0)	
More than 3 times	**9 (6.3)**	**3 (1.5)**	
Interval to last CS (years, mean/SD)	7.5 ± 4.4	7.3 ± 3.7	.928
History of hysteroscopic surgery, n (%)	8 (6)	1 (0.5)	.**005**
Pregnancy mood swings, n (%)			
No	57 (40)	92 (46)	.511
Mild	80 (56)	100 (50)	
Medium	5 (3.5)	4 (2)	
Severe	1 (0.7)	3 (1.5)	

*Note*: Values in bold indicate statistical significance (*P* < .05).

**Table 3:** The Association of the Number of Previous Cesarean Section(s) and Subtypes of CSP

Number of Previous CS	Type 1 CSP (n = 143), n (%)	Type 2 CSP (n = 200), n (%)	Odds Ratio (95% CI)
≤2	134 (93.7)	197 (98.5)	4.410 (1.184, 15.31, *P* = .032)
≥3	9 (6.3)	3 (1.5)	

Changes in hormone levels during pregnancy, especially in the early stage of pregnancy, are associated with pregnancy mood swings. We next compared the pregnancy mood swings between women with type 1 and type 2 CSP and found that there were no statistical significances in the pregnancy reactions between women with type 1 and type 2 CSP (*P* = .511).

## Discussion

In this study with large sample size, we found that maternal age at the time of diagnosis of CSP, maternal BMI, the number of previous CSPs, gravidity, parity, and the number of elective abortions were not associated with the development of type 1 or type 2 CSP. In addition, the interval between the last cesarean section and the current pregnancy and the levels of serum β‐hCG were also not associated with the development of different types of CSP. However, women with more than 3 times of prior cesarean section had an increased risk of developing type 1 CSP. Additionally, a higher number of women with type 2 CSP had vaginal bleeding before diagnosis.

CSP refers to a gestational sac fully or partially implanted in the site of a scar from a previous cesarean section.^5^ Identifying risk factors specific to each subtype could enhance our ability to predict CSP type, tailor interventions, and potentially improve outcomes. A key difference between type 1 (endogenic) and type 2 (exogenic) CSP is the implantation site of the gestational sac. Although some studies used 3 types of classification, both type 2 and type 3 share similar features of exogenic CSP. Additionally, these types often require similar management approaches, particularly in terms of surgical intervention. In type 1 CSP, the gestational sac is implanted on the scar site, whereas in type 2 CSP, it is deeply embedded within the scar and the surrounding myometrium. This distinction has led to speculation that women with multiple cesarean sections, who typically have more extensive scar tissue, may be at increased risk of developing type 2 CSP. However, in our study, we found that women with type 1 CSP had significantly more than 3 prior cesarean sections compared to those with type 2 CSP, but there was no association in CSP women with 2 prior cesarean sections between the 2 subtypes. The exact reason for this unexpected finding remains unclear, but it suggests that multiple factors, aside from the small sample size in CSP women with more than 3 prior cesarean sections, may contribute to the development of CSP subtypes.

Hysteroscopic surgery could “damage” the surface of the endometrium, resulting in extensive scar tissue. Our current study also found the association of hysteroscopic surgery with the developing type 1 CSP. However, due to the small sample size in CSP women with 3 times prior cesarean sections or CSP women with a history of hysteroscopic surgery, this association should be further investigated.

There is a lack of evidence in the current literature regarding whether hormonal levels could influence the type of CSP that develops. However, hormonal imbalances might affect how the fertilized egg implants in the uterine tissue. CSP is associated with low or absent β‐hCG levels,^18^ and lower estrogen levels are associated with an increased risk of vaginal bleeding.^19^, ^20^ Our current study does not have data on sex hormone levels, such as estrogen and progesterone, in women with CSP, as these tests are not routinely performed. Nonetheless, hormonal changes during pregnancy, particularly in its early stages, are known to influence mood swings (https://americanpregnancy.org/healthy‐pregnancy/pregnancy‐health‐wellness/mood‐swings‐during‐pregnancy). Estrogen, for instance, is active in regions of the brain that regulates mood.^21^ To explore this indirectly, we compared pregnancy‐related mood swings between type 1 and type 2 CSP, using these observations as a proxy for sex hormone levels. Our data showed no statistical significance in early pregnancy mood reactions between women with type 1 and type 2 CSP. In contrast, a significantly higher number of women with type 2 CSP experienced vaginal bleeding compared to those with type 1 CSP. This finding suggests that vaginal bleeding may be associated with type 2 CSP and indirectly indicates a potential link between lower estrogen levels and the development of this subtype. However, this association should be interpreted as part of a broader pattern of clinical differences between the subtypes, rather than as a definitive predictive factor. Reliable CSP classification will likely require a combination of clinical, imaging, and potentially molecular markers. Further studies with larger sample sizes and prospective designs are needed to validate this finding and determine its role in clinical decision‐making.

The interval between the previous cesarean sections and the current pregnancy (CSP) could influence the type of CSP. The short interval could result in the incompletion of scar tissue healing, which may lead to the development of type 1 CSP. In this study, we did not see a difference in the interval between the last cesarean section and the current pregnancy between women with type 1 and type 2 CSP. This could be because the interval time was around 7 years in both groups, suggesting the scar tissue is recovered.

The large sample size included in this study was one strength. However, this study has several limitations. In our study, we focused on a straightforward comparison of the clinical differences between type 1 and type 2 CSP, such as parity, gravidity, surgical abortion history, previous CSP, and the interval to the last cesarean section. While we recognize that confounding factors may influence the associations we observed, our study was designed as a descriptive analysis to identify potential differences between the 2 CSP subtypes based on clinical characteristics. Advanced statistical techniques such as pairwise matching, propensity score analysis, or multivariate analysis could certainly help control for confounding variables and further validate the findings. Future studies employing these methods would be beneficial to more rigorously control for confounding factors and enhance the robustness of the conclusions. Additionally, the sample size of patients with more than 3 cesarean sections in both groups was relatively small. Future studies with large sample sizes are required to confirm our findings.

Analyzing clinical risk factors of developing type 1 and type 2 CSP is essential, as understanding the differences and potential factors impacting the development of each type can contribute to improved clinical management and outcomes. In conclusion, our study with a large sample size found that both types of CSP appear to share common risk factors, and there were no differences in the clinical risk factors for distinguishing the 2 types of CSP. However, multiple cesarean sections (more than 3 times) are associated with the developing type 1 CSP, and vaginal bleeding in the early stage of pregnancy may be related to the developing type 2 CSP.

## Data Availability

The data that support the findings of this study are available from the corresponding author upon reasonable request.
